# COVID-19 Among Workers in the Seafood Processing Industry: Implications for Prevention Measures — Alaska, March–October 2020

**DOI:** 10.15585/mmwr.mm7017a4

**Published:** 2021-04-30

**Authors:** Kimberly A. Porter, Maya Ramaswamy, Thomas Koloski, Louisa Castrodale, Joseph McLaughlin

**Affiliations:** ^1^Division of State and Local Readiness, Center for Preparedness and Response, CDC; ^2^CDC COVID-19 Response Team; ^3^Alaska Department of Military and Veterans Affairs, ^4^Alaska Division of Public Health.

Large COVID-19 outbreaks have occurred in high-density workplaces, such as food processing facilities (*1*). Alaska’s seafood processing industry attracts approximately 18,000 out-of-state workers annually (*2*). Many of the state’s seafood processing facilities are located in remote areas with limited health care capacity. On March 23, 2020, the governor of Alaska issued a COVID-19 health mandate (HM10) to address health concerns related to the impending influx of workers amid the COVID-19 pandemic (*3*). HM10 required employers bringing critical infrastructure (essential) workers into Alaska to submit a Community Workforce Protective Plan.* On May 15, 2020, Appendix 1 was added to the mandate, which outlined specific requirements for seafood processors, to reduce the risk for transmission of SARS-CoV-2, the virus that causes COVID-19, in these high-density workplaces (*4*). These requirements included measures to prevent introduction of SARS-CoV-2 into the workplace, including testing of incoming workers and a 14-day entry quarantine before workers could enter nonquarantine residences. After 13 COVID-19 outbreaks in Alaska seafood processing facilities and on processing vessels during summer and early fall 2020, State of Alaska personnel and CDC field assignees reviewed the state’s seafood processing–associated cases. Requirements were amended in November 2020 to address gaps in COVID-19 prevention. These revised requirements included restricting quarantine groups to ≤10 persons, pretransfer testing, and serial testing (*5*). Vaccination of this essential workforce is important (*6*); until high vaccination coverage rates are achieved, other mitigation strategies are needed in this high-risk setting. Updating industry guidance will be important as more information becomes available.

On May 15, 2020, the state issued HM10 Appendix 1, detailing three entry quarantine options for onshore seafood processors: 1) quarantine workers for 14 days before travel to Alaska (pretravel quarantine), 2) quarantine workers in an Alaskan community with a general acute care or critical access hospital (midtravel quarantine), or 3) quarantine workers at the destination community after arrival (posttravel quarantine) (Table 1). These options also included requirements for safe transit^†^ (e.g., chartered air travel) and for each worker to receive one or more (depending on the quarantine option selected) negative reverse transcription–polymerase chain reaction (RT-PCR) tests for SARS-CoV-2. A separate but similar set of options was available for workers boarding processing vessels (*4*). HM10 Appendix 1 also included a requirement for using safe transit during transfer of workers between facilities (*4*).

**TABLE 1:** Entry quarantine options for onshore seafood processors under initial Alaska COVID-19 health mandate 10, appendix 1*

Option	Quarantine	Testing	Transit	Destination community
Pretravel quarantine	Workers observed a 14-day monitored quarantine period outside of Alaska.	RT-PCR^†^ test was done within 48 hours before beginning travel to Alaska.	Safe transit^§^ was used for all travel to the processing facility in the destination community on a chartered aircraft, ground vehicle, or vessel.	Workers entered the nonquarantine quarters upon arrival and started work alongside workers who had completed quarantine.
Midtravel quarantine	Workers traveled to Alaska to observe a 14-day monitored quarantine period in temporary lodging in a large community with a general acute care or critical access hospital.	RT-PCR test was done within 48 hours before beginning onward travel to the destination community.	All travel from the quarantine location to the processing facility in the destination community was accomplished via safe transit.	Workers entered the nonquarantine quarters upon arrival and started work alongside workers who had completed quarantine.
Posttravel quarantine	Workers traveled to their final destination community in Alaska to observe a 14-day quarantine, housed individually or in a quarantine group (workers living or working in close proximity were assigned to a quarantine group and completed quarantine together).	RT-PCR test was done before entering monitored quarantine lodging. (Another test was done at day 6 and within 48 hours of completion of quarantine as supplies allowed.)	Travel to the destination community was done via commercial transit.	Workers were permitted to work during their 14-day quarantine period under specific circumstances**.^¶^**

**Abbreviation:** RT-PCR = reverse transcription–polymerase chain reaction.

* Issued on May 15, 2020 (https://covid19.alaska.gov/wp-content/uploads/2020/05/COVID-MANDATE-10-Appendix-01.pdf).

^†^ Using a Food and Drug Administration–authorized test.

^§^ Safe transit is a mode of transportation in which all employees have completed quarantine and testing requirements, are not interacting with any populations whose quarantine and testing status is unknown, and are physical distancing, using appropriate personal protective equipment to isolate the travelers from the vehicle crew, or both.

^¶^ Specific circumstances refers to a situation in which tasks can be conducted while maintaining 6-ft physical distancing measures, or using physical barriers and personal protective equipment to separate workers from all other workers outside of their quarantine group.

After 13 COVID-19 outbreaks occurred in seafood processing facilities and on processing vessels through early fall 2020, Alaska-based CDC field assignees assisted State of Alaska personnel with revising HM10 Appendix 1 by reviewing data from investigations of the state’s laboratory-confirmed SARS-CoV-2^§^ cases that occurred during March 1–October 13, 2020. Seafood processing–associated cases were identified by querying the state’s reportable disease database and searching records obtained during outbreak investigations. In addition, the number of cases identified under certain circumstances (e.g., cases identified during entry quarantine or after workers were transferred from one facility to another) was evaluated using detailed notes from public health investigations. This activity was reviewed by CDC and was conducted consistent with applicable federal law and CDC policy.^¶^

During the period reviewed, 677 cases of SARS-CoV-2 infection were identified among seafood processing industry workers (Figure). Among these, 132 cases were either independent cases (i.e., did not result in transmission to another person) during entry quarantine or were part of a cluster of infections within an entry quarantine group (i.e., a group of workers living and working solely with each other). Among the remaining cases, 539 were either part of outbreaks that spread beyond an entry quarantine group or included persons outside of entry quarantine, including local workers; six cases were not classified because of insufficient information. 

**FIGURE:**
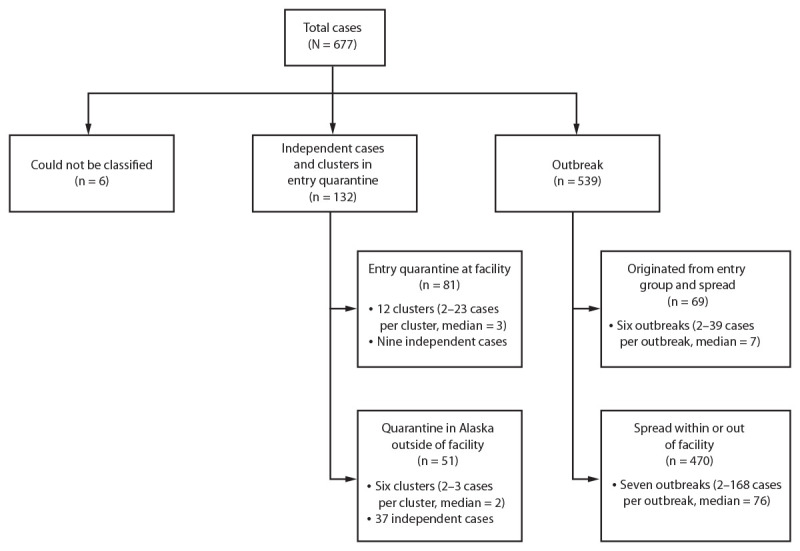
Laboratory-confirmed cases*^,^^†^ of COVID-19 associated with the seafood processing industry^§^ — Alaska, March 1–October 13, 2020 * Clusters include those determined to include person-to-person transmission within an entry quarantine group. ^†^ Independent cases were not known to have transmitted SARS-CoV-2 to others. ^§^ The source of the spread within or outside of facility was unknown.

Among the 132 cases that were independent or part of a cluster within an entry quarantine group, 81 cases (61%) occurred in workers quarantined at an onshore processing facility; 72 (89%) of these cases were part of a cluster. Twelve distinct clusters of 2–23 cases (median = 3 cases), were identified at facilities conducting entry quarantine in the destination community (Figure). Persons completing entry quarantine at the processing facility in the destination community were usually housed in groups and allowed to work if they were able to maintain a distance of 6 ft or use physical barriers and personal protective equipment to separate themselves from other workers outside of their quarantine group. Although persons with positive SARS-CoV-2 RT-PCR test results were removed from these groups for isolation once they were identified, transmission within the entry quarantine group occurred. The remaining 51 (39%) cases occurred in workers quarantined off-site in Alaska; 37 (73%) of these were independent cases with no known onward transmission.

Thirteen distinct outbreaks identified in onshore facilities or on vessels involved persons who had either completed entry quarantine, were in a different entry quarantine group, or who were local workers. Attack rates in onshore facilities and vessels ranged from <5%–75%. Six outbreaks (range = 2–39 cases, median = 7 cases) appeared to have originated in an entry quarantine group and then spread (Figure). The remaining seven outbreaks (range = 2–168 cases, median = 76 cases) were of unknown origin; these outbreaks were responsible for 470 (87%) of the 539 outbreak-associated cases. One outbreak of 39 cases was the result of a midseason crew transfer in which persons previously not known to be infected were moved via safe transit from a facility that had experienced an outbreak to a closed campus (i.e., a facility with no or limited interaction with local persons) where the workers had previously received negative test results. A separate outbreak of 168 cases was identified only after an employee sought care for a non–COVID-19 medical issue and was screened as part of that visit.

As a result of the large number of cases that occurred among workers outside entry quarantine, additional prevention measures were developed to further reduce risk (Table 2). These were reflected in revised requirements implemented in November 2020 (*5*).

**TABLE 2:** Selected requirements from Alaska COVID-19 health mandate 10, appendix 1* and Alaska health order 5 (revised appendix 1)^†^

Protective measure	Original requirements	Revised requirements
Posttravel entry quarantine	Entry quarantine groups were kept “as small as possible” and allowed to work during quarantine under specific circumstances.^§^	Entry quarantine groups were ≤10 persons and prohibited from working during quarantine.
Midseason transfers	Safe transit^¶ ^was used for all travel from one location to another; if not available and transferring workers had to travel within 6 ft for >10 min with persons whose quarantine status was not known, transferring workers had to repeat their quarantine period at the new location, with RT-PCR** testing on day 6 and within 48 hours before being released from quarantine.	Pretransfer testing was also required for persons leaving a vessel or onshore facility that had experienced an outbreak.
Serial testing	Not included	Serial testing was required. Guidance for the frequency of testing was based on risk category^††^ and facility type (e.g., open or closed campuses).
Response to a positive worker	Not included	Notifying public health, isolating confirmed cases, and quarantining close contacts explicitly required (with detailed instructions provided), as was a requirement to develop an outbreak contingency plan.
Daily symptom screening	Only required during the entry quarantine	Daily symptom screening of workers required throughout the season.

**Abbreviation:** RT-PCR = reverse transcription–polymerase chain reaction.

* Issued on May 15, 2020 (https://covid19.alaska.gov/wp-content/uploads/2020/05/COVID-MANDATE-10-Appendix-01.pdf).

^†^ Issued on November 16, 2020 (https://covid19.alaska.gov/wp-content/uploads/2020/12/Outbreak-Health-Order-No-5-Appendix-01-Enhanced-Protective-Measures-for-Seafood-Processing-Workers-DD3.pdf).

^§^ Specific circumstances refers to a situation in which tasks can be conducted while maintaining 6-ft physical distancing measures, or using physical barriers and personal protective equipment (PPE) to separate workers from all other workers outside of their quarantine group.

^¶^ Safe transit is a mode of transportation in which all employees have completed quarantine and testing requirements, are not interacting with any populations whose quarantine and testing status is unknown, and are physical distancing, using appropriate PPE to isolate the travelers from the vehicle crew, or both.

** Using a Food and Drug Administration–authorized test.

^††^ Risk categories were based on the local alert level (if available) or the alert level at the community school. The community-level indicators used by local jurisdictions and schools to assign their alert level varied by locality. Companies were asked to use the alert level in combination with the timing of the arrival of new workers to determine their risk category.

## Discussion

After review of the state’s seafood processing–associated cases, a revision of the required measures went into effect on November 16, 2020 to address gaps in COVID-19 prevention (*5*). Introduction of the virus into remote areas was likely reduced when the 51 persons with positive SARS-CoV-2 test results were identified during entry quarantine outside of the facility and thus completed isolation off-site. Entry quarantine at a processing facility in the destination community was less effective and led to clusters within entry quarantine groups. The revised requirements restricted the size of quarantine groups to ≤10 persons; HM10 Appendix 1 had included guidance to keep the groups “as small as possible.” The revision also eliminated the option for working during entry quarantine.

Expanding the scope of the required measures was also necessary. The outbreak that occurred after a transfer of crew from one processing facility to another indicated that recommending safe transit for midseason crew changes was inadequate for eliminating the risk for interfacility transmission. A pretransfer testing requirement was included in the revised measures to reduce the risk of unintentional movement of infected persons. Another outbreak was identified only after a worker who was seeking non–COVID-19-related health care was tested, indicating that identification of outbreaks was not always timely. Because serial testing of all workers throughout the season might be a more effective strategy to identify outbreaks earlier, a requirement for serial testing was included in the revised measures. 

The findings in this report are subject to at least four limitations. First, case counts were based on surveillance data and might be subject to small discrepancies. Second, a comparison before and after implementation of the revised requirements was not possible because the initial set of required measures was issued early in the seafood processing season that took place during the summer months. Third, the lack of precise denominators restricted analysis of the overall rate of disease among seafood processing workers. Finally, quantifying the size of outbreaks was often challenging because testing strategies conducted after cases were identified varied considerably among facilities, which likely affected case finding. For example, in response to an outbreak identified at one facility, the company elected to conduct multiple rounds of mass testing and ultimately determined that 168 (61%) workers were infected. Another company that identified cases conducted little additional testing, and fewer than 10 cases among approximately 500 workers were ultimately identified.

These findings suggest that requiring entry testing and quarantine might have reduced importations of SARS-CoV-2 into remote seafood processing facilities and vessels. Incorporating additional measures, such as serial testing and restricting work during quarantine, might further reduce the risk to seafood processing workers and the communities in which they work. Vaccination of this essential workforce is important (*6*) and underway. Updated guidance for the industry will be needed as more is learned about how mitigation strategies might change in high-density workplaces when high vaccination coverage levels are achieved.

**Summary**

**What is already known about this topic?**

Large outbreaks of COVID-19 have occurred in high-density workplaces. In May 2020, Alaska mandated prevention measures in the seafood processing industry.

**What is added by this report?**

A review of COVID-19 cases and outbreaks in this industry found that entry quarantine and testing might have reduced introduction of the virus to seafood processing facilities and vessels. The review also identified gaps in the required COVID-19 prevention strategies. Findings were used to revise requirements, which included the addition of serial testing.

**What are the implications for public health practice?**

Until high vaccination coverage rates are achieved among the seafood processing workforce, rigorous mitigation strategies are needed to prevent and control outbreaks in this high-risk setting.

## Footnotes

*
https://covid19.alaska.gov/unified-command/protective-plans/

† Safe transit is a mode of transportation in which all employees have completed quarantine and testing requirements, are not interacting with any populations whose quarantine and testing status is unknown, and are physical distancing, using appropriate personal protective equipment to isolate the travelers from the vehicle crew, or both.

§ Laboratory confirmation requires detection of SARS-CoV-2 RNA in a clinical specimen using a molecular amplification detection test (https://wwwn.cdc.gov/nndss/conditions/coronavirus-disease-2019-covid-19/case-definition/2020/).

¶ 45 C.F.R. part 46.102(l)(2), 21 C.F.R. part 56; 42 U.S.C. Sect. 241(d); 5 U.S.C. Sect. 552a; 44 U.S.C. Sect. 3501 et seq.
